# Comparison of kinesio taping and sham taping in patients with chronic low back pain: A protocol of randomized controlled trial

**DOI:** 10.1097/MD.0000000000023042

**Published:** 2020-11-20

**Authors:** Dongliang Wang, Siqing Wang, Kun Lu, Yongming Sun

**Affiliations:** a Department of Spine Surgery, Yancheng First People's Hospital of Jiangsu Province; b Department of Orthopedics, Second Affiliated Hospital of Suzhou University, China.

**Keywords:** chronic low back pain, kinesio tape, pain control, study protocol

## Abstract

**Background:**

Chronic low back pain (CLBP) is a clinical condition characterized by moderate to severe pain in the lower spine that severely affects the patient's life experience and leads to disability and absenteeism. In the past few years, kinesio tape (KT) have been utilized by physiotherapists as a relatively novel band-aid method to reduce the pain of musculoskeletal disorders. Therefore, in this particular study, we intended to search the effects of KT and sham KT on pain, lumbar range of motion, and disability for CLBP.

**Methods:**

The present study was experimented in a physiotherapy clinic in the Yancheng First People's Hospital of Jiangsu Province. The study design was a randomized, double-blinded clinical trial. Inclusion criteria for the study were the followings: chief complaint pain in the area between 12 ribs and hip creases with or without leg pain; ages ranges from 18 to 65; low back pain lasts <6 weeks; and at any rate medium pain intensity (pain score ≥4). Participants were randomly allocated to 1 of 2 parallel combinations to receive either therapeutic KT or sham KT. Patients were assessed at baseline, at the end of the 12-day intervention, and at 4 weeks of follow-up. The main result measure was pain intensity using a numerical rating scale (NRS), and the secondary outcome measure was lumbar lateral flexion activity, Oswestry Disability Index (ODI), and adverse effects including allergic reactions or skin problems.

**Conclusions:**

The results of this study will provide new information about the usefulness of KT as an additional component of a guideline-endorsed physiotherapy program in patients with chronic nonspecific low back pain.

**Trial registration:**

This study protocol was registered in Research Registry (researchregistry6070).

## 1 Introduction

Chronic low back pain (CLBP) is a clinical condition characterized by moderate to severe pain in the lower spine that severely affects the patient's life experience and leads to disability and absenteeism.^[1–4]^ Studies have shown that the number of people accessing health care for low back pain has increased in recent years, and that patients with low back pain seek more treatment than those with acute low back pain.^[5–7]^ Many conservative treatments for pain, such as physical therapy and nonsteroidal anti-inflammatory drugs have been experimented, but the best ones are still being debated.^[8,9]^

In the past few years, kinesio tape (KT) have been utilized by physiotherapists as a relatively novel band-aid method to reduce the pain of musculoskeletal disorders.^[10–12]^ KT is an elastic bonding material with high tensile capacity, which ensures the free movement of the application area without the need for chemicals.^[13]^ KT can be extended to 140% of the original length, providing a good range of motion compared with other types of tape. Studies have shown that KT improves blood and lymph circulation, mitigates pain, adjusts joints, and relives muscle tension.^[14–17]^ Although the effect of KT on pain is unclear, KT may provide afferent stimuli that promote pain inhibition mechanisms and pain relief.

Past studies have reported debatable results in favor of KT as an appropriate intervention for CLBP patients. So far, the short-dated effects of KT on various musculoskeletal problems such as knee osteoarthritis remain unclear. Sheng et al^[18]^ conducted a meta-analysis in order to compare the efficacy of KT on CLBP with that of other general physical therapies and confirm its positive effects. However, the other reviews could not reach conclusive evidence of bright side of KT.^[19–21]^ Therefore, in this particular study, we intended to search the effects of KT and sham KT on pain, lumbar range of motion, and disability for CLBP.

## 2 Material and method

### 2.1 Study design and patients

The present study was experimented in a physiotherapy clinic in the Yancheng First People's Hospital of Jiangsu Province. The study design was a randomized, double-blinded clinical trial. This study program was reviewed and approved by the institutional review committee of the Yancheng First People's Hospital of Jiangsu Province (1002320). Written consent was obtained from the participants before starting the study. Furthermore, the program of the study was registered in Research Registry (researchregistry6070). Between October 2020 and October 2021, our agency will evaluate 100 eligible CLBP patients.

Inclusion criteria for the study were the followings: chief complaint pain in the area between 12 ribs and hip creases with or without leg pain; ages ranges from 18 to 65; low back pain lasts <6 weeks; and at any rate medium pain intensity (pain score ≥4). Exclusion criteria included: known or suspected severe spinal lesions; spinal surgery within the first 6 months; severe complications preventing prescription paracetamol; and physical therapy for lower back pain for the past 6 months.

### 2.2 Randomization

Participants were randomly allocated to 1 of 2 parallel combinations to receive either therapeutic KT or sham KT (ratio: 1:1). To allocate the participants, the “Research Randomiser,” which is an online randomization web service, was used. Simple randomization procedures were conducted, and sequentially numbered index cards with the random assignment were prepared by an investigator with no clinical involvement in the study. The index cards were folded and placed in sealed, opaque envelopes. Then, the blind investigator opened each envelope and allocated the participants to the KT or placebo taping group according to the selected index card.

### 2.3 Interventions or controls

In the intervention group, the most suffering area of the low back applied KT. The KT technique: Curetape (TapeConcept Ltd., Larnaca, Cyprus) was utilized in this study. Before using KT, clean the skin on the lower back with an alcohol swab to make sure no lotion or grease. Excessive hair must be shaved for better outcomes and to reduce pain when removing tape. In the control group, the sham tape was used with the same method.

The tape was applied 3 times a week at 1-day intervals after the previous tape was removed in each session. After every tape removal, the therapist checked for skin sensitivity reactions. Overall, 9 taping sessions were used in all 3 groups. Treatment was continued for 3 weeks.

### 2.4 Outcomes and measures

After allocation, baseline measures are taken. All the data were collected by the researchers, who were unaware of the assigned group of patients. Patients were assessed at baseline, at the end of the 12-day intervention, and at 4 weeks of follow-up. The main result measure was pain intensity using a numerical rating scale (NRS), and the secondary outcome measure was lumbar lateral flexion activity, Oswestry Disability Index (ODI), and adverse effects including allergic reactions or skin problems. The NRS of people are assessed for pain on an 11-point scale ranging from zero (for “no pain”) to 10 (for “most severe pain possible”). The ODI questionnaire included 10 items related to daily life activities (such as personal care, elevations, walking, sitting, sleeping, social life, travel, work) restrictions. Each question consists of 6 possible responses on a scale of “5” or “completely disabled” with a minimum of “0” or “no disability.”

### 2.5 Sample size calculation

The study was depicted to find out an intergroup difference of 1 point in pain intensity as measured by the NRS, with an estimated standard deviation of 1.84, an intergroup difference of 18 points and an estimated standard deviation of 12 points for disability as measured by the ODI questionnaire. The other specifications were: 80% power, 5% alpha, subsequent losses up to 15%. Therefore, a total of 100 participants (50 per group) were enlisted for this research. Estimates used in sample size calculations were lower than those recommended as minimum clinically significant differences to improve the accuracy of estimates of intervention effectiveness.

### 2.6 Statistical analysis

Mean and standard deviation are descriptive statistics. Kolmogorov–Smirnov test was utilized for the normality of all data. Paired sample *t* test was utilized to compute the value difference before and after treatment. To compare the variances between the 2 groups, we utilized the student's *t* test. The significance level of *P* < .05 was accepted. Every analysis was performed using the PASW for Windows 20.0 software program (SPSS Inc., Chicago, IL).

## 3 Discussion

There is a wealth of clinical evidence, including 2 meta-analyses, 4 systematic reviews of musculoskeletal conditions, and a consistent conclusion about low back pain that KT is no better for these patients than placebo. At present, there is not enough evidence to prove the effect of exercise tablet on postural control and balance in patients with chronic low back pain. Therefore, in this particular study, we intended to search the effects of KT and sham KT on pain, lumbar range of motion, and disability for CLBP.

## Author contributions

**Conceptualization:** Dongliang Wang.

**Data curation:** Dongliang Wang, Siqing Wang.

**Formal analysis:** Dongliang Wang, Siqing Wang.

**Funding acquisition:** Yongming Sun.

**Investigation:** Dongliang Wang, Siqing Wang.

**Methodology:** Dongliang Wang, Siqing Wang.

**Project administration:** Kun Lu, Yongming Sun.

**Resources:** Kun Lu.

**Software:** Siqing Wang.

**Supervision:** Yongming Sun.

**Validation:** Siqing Wang.

**Visualization:** Dongliang Wang.

**Writing – original draft:** Dongliang Wang.

**Writing – review & editing:** Yongming Sun, Kun Lu.
